# Atypical Multisensory Integration and the Temporal Binding Window in Autism Spectrum Disorder

**DOI:** 10.1007/s10803-020-04452-0

**Published:** 2020-03-24

**Authors:** Sayaka Kawakami, Shota Uono, Sadao Otsuka, Sayaka Yoshimura, Shuo Zhao, Motomi Toichi

**Affiliations:** 1 Faculty of Human Health Sciences, Graduate School of Medicine, Kyoto University, 53 Shogoin Kawahara-cho, Sakyo-ku, Kyoto, 606-8507 Japan; 2 Department of Neurodevelopmental Psychiatry, Habilitation and Rehabilitation, Faculty of Human Health Sciences, Graduate School of Medicine, Kyoto University, 53 Shogoin Kawahara-cho, Sakyo-ku, Kyoto, 606-8507 Japan; 3 Department of Psychiatry, Graduate School of Medicine, Kyoto University, 54 Shogoin-kawahara-cho, Sakyo-ku, Kyoto, 606-8507 Japan; 4 The Organization for Promoting Neurodevelopmental Disorder Research, 40 Shogoin-Sannocho, Sakyo-ku, Kyoto, 606-8392 Japan; 5 School of Psycholgy, Shenzhen Key Laboratory of Affective and Social Neurosience, Shenzhen University, Shenzhen, China

**Keywords:** Autism spectrum disorder (ASD), Sensory processing, Audiovisual, Temporal processing, Multisensory integration, Symptom

## Abstract

The present study examined the relationship between multisensory integration and the temporal binding window (TBW) for multisensory processing in adults with Autism spectrum disorder (ASD). The ASD group was less likely than the typically developing group to perceive an illusory flash induced by multisensory integration during a sound-induced flash illusion (SIFI) task. Although both groups showed comparable TBWs during the multisensory temporal order judgment task, correlation analyses and Bayes factors provided moderate evidence that the reduced SIFI susceptibility was associated with the narrow TBW in the ASD group. These results suggest that the individuals with ASD exhibited atypical multisensory integration and that individual differences in the efficacy of this process might be affected by the temporal processing of multisensory information.

Autism spectrum disorder (ASD) is a neurodevelopmental disorder characterized by deficits in social communication, repetitive behaviors, and restricted interests (American Psychiatric Association [APA] 2013). Although previous research has examined the possible causal role of social and cognitive dysfunction in the social communication difficulties of this group (cf. Otsuka et al. 2017), more recent studies have proposed that these dysfunctions are influenced and/or induced by alterations in lower-order fundamental functions (Baum et al. 2015; Elsabbagh et al. 2016). Atypical multisensory processing is among the candidate fundamental functions involved in the social communication difficulties in ASD. Multisensory processing confers many social and cognitive advantages. For example, the ability to detect and react to targets is enhanced by multisensory information (Brandwein et al. 2013; Juan et al. 2017). Observations of how a speaker’s mouth moves (i.e., visual information) help us understand what the speaker is saying (i.e., auditory information; Ross et al. 2007), and interpretations of facial expressions are affected by simultaneous emotional vocal cues (Campanella and Balin 2007; de Gelder and Vroomen 2000). In addition to the perceptual and recognition advantages, multisensory information contributes to learning (Shams and Seitz 2008); for example, auditory information facilitates visual learning (Seitz et al. 2006). Indeed, these studies suggest that perception, recognition and learning of social information, and high-level social–cognitive functioning may be affected by multisensory processing alterations.

The sound-induced flash illusion (SIFI) task has been used to explore the ability of people with ASD to engage in multisensory processing. The SIFI is a phenomenon in which auditory stimuli affect the perception of a visual stimulus (Shams et al. 2000); when one flash is presented along with two or more beeps, the auditory information induces the perception of illusory flashes. The SIFI task employed in the present study used non-social stimuli (e.g., flashes and beeps) and has the advantage of allowing for the evaluation of multisensory processing itself whereas other illusion-exploring multisensory integration phenomena that include social stimuli (e.g., the McGurk effect) might be affected by the difficulties experienced by individuals with ASD when processing social information. Previous studies have shown that individuals with ASD and typically developing (TD) individuals exhibit comparable multisensory integration capabilities for visual and tactile modalities (Poole et al. 2015) and, similarly, other studies have reported that individuals with and without ASD perform similarly on the SIFI (van der Smagt et al. 2007; Keane et al. 2010; Stevenson et al. 2018a, b). However, other studies have reported inconsistent findings of atypical SIFI perceptions in ASD that suggest problems with audio–visual integration. Foss-Feig et al. (2010) reported that children with ASD were more likely to perceive an illusory flash than were TD children. However, Stevenson et al. (2014) found that children with ASD were less likely to perceive the SIFI than were TD children. Kawakami et al. (2018) also demonstrated that young adults with high levels of autistic traits in a general population were less likely to perceive the SIFI than those with low levels of such traits. In addition to differences in the demographic characteristics of the clinical population (e.g., intellectual ability, age, and gender), methodological differences in the experimental design used might have affected the results regarding SIFI susceptibility in different groups. The former study presented a single flash with two to four beeps in the illusory trials and reported increased SIFI perception in children with ASD (Foss-Feig et al. 2010). In contrast, the latter studies presented a single flash with two beeps in the illusory trials and varied the stimulus onset asynchrony (SOA) between beeps with and without a flash, and reported that children with ASD (Stevenson et al. 2014) and TD adults with high levels of autistic traits (Kawakami et al. 2018) are less likely to perceive the SIFI. The present study tried to replicate the reduced perception of the SIFI in adults with ASD by presenting a single flash with two beeps in illusory trials (Kawakami et al. 2018; Stevenson et al. 2014).

Individual differences in multisensory integration (e.g., SIFI perception) can be at least partially explained by differences in temporal processing. Even when two or more types of information are not actually synchronous, people can perceive those pieces of information as being synchronized within a specific range of the temporal binding window (TBW). Previous studies have shown that TBW size is an important determinant of multisensory integration (Wallace and Stevenson 2014; Stevenson et al. 2018b). For example, Stevenson et al. (2012) investigated individual differences in the size of the TBW in a general population and reported that individuals with a narrower TBW (i.e., high susceptibility to a time lag) are less likely to perceive the SIFI, which is an index of audio–visual integration. A recent study showed that TBW narrowing through training is associated with reduced SIFI susceptibility, suggesting the importance of the TBW in multisensory integration (Setti et al. 2014). In addition, studies have reported that TBW size is associated with cognitive functioning (Zmigrod and Zmigrod 2016) and the severity of clinically important features (Kawakami et al. 2018 for autistic traits; Stevenson et al. 2017 for hallucinations). These reports suggest that adaptive social and cognitive functioning depend on the appropriate size of the TBW such that a too-wide TBW is associated with a tendency toward integrating unrelated information whereas a too-narrow TBW is related to difficulties with integrating sensory inputs.

Previous studies have investigated TBWs in terms of multisensory integration using a temporal order judgment (TOJ) task (Jaśkowski et al. 1990; Rutschmann and Link 2016). In this multisensory TOJ task, participants are asked to judge whether a stimulus (e.g., a beep) appeared “early” or “late” compared to another stimulus (e.g., a flash) when the visual and auditory stimuli were presented at variable SOAs. The TBW was defined as the time interval at which the participant could not accurately judge the presentation order of the flash and sound (e.g., de Boer-Schellekens 2013). Individuals who are able to judge the order of the visual and auditory stimuli correctly under a short SOA condition have a narrow TBW. However, there have been conflicting findings about differences in the TBW between ASD and TD groups in the multisensory TOJ task (cf. Stevenson et al. 2016). When using non-social stimuli, some studies have reported no significant differences between ASD and TD groups in the size of the TBW (Poole et al. 2017; Stevenson et al. 2018b) whereas other studies found that individuals with ASD have a wider TBW compared to TD individuals (Kwakye et al. 2011; de Boer-Schellekens et al. 2013). On the other hand, Kawakami et al. (2018) reported that TD adults who have higher autistic traits are more sensitive to time lags between auditory and visual stimuli (i.e., narrow TBW). However, it remains unclear whether individuals with ASD would show an atypical TBW size for integrating multisensory information in the TOJ task even though two recent studies relevant to ASD reported a strong relationship between TBW size and multisensory processing. Kawakami et al. (2018) demonstrated that TD adults with high levels of autistic traits have a narrow TBW that is associated with a reduced ability to integrate asynchronous and non-social multisensory information (i.e., low SIFI susceptibility). Stevenson et al. (2018b) found that a wider TBW is related to a reduced ability to integrate synchronous and social multisensory information (i.e., McGurk effect) in children with ASD. Based on these findings, it remains unclear whether adults with ASD differ from others regarding the size of the TBW and, if so, how this difference affects multisensory integration.

Thus, the present study evaluated multisensory integration and the temporal processing of high-functioning adults with ASD to address several unresolved issues. First, although the relationship between TBW size and multisensory integration has been reported in a general population (Stevenson et al. 2012, 2018b; Kawakami et al. 2018), it is unclear whether the same relationship holds in adults with ASD. Similar to a previous study (Kawakami et al. 2018), the present study employed the TOJ and SIFI tasks as measures of TBW size and the ability to engage in multisensory integration, respectively. In the TOJ task, the temporal range within which participants could not discriminate the temporal order of two stimuli was calculated to represent the size of the TBW. In the SIFI task, we assessed the frequency with which the SIFI was perceived as an indicator of multisensory integration. The results of these two tasks were compared between the ASD and TD groups after controlling for age, sex, and intelligence quotient (IQ) and the relationships between TBW size and SIFI frequency were evaluated. Second, although some studies using other tasks containing social information have reported that altered fundamental processing (i.e., temporal and multisensory processing) affects the severity of ASD (Smith et al. 2017; Mongillo et al. 2008), it is unclear whether the multisensory processing performance assessed by non-social tasks (i.e., the SIFI and the flash–beep TOJ) can explain individual differences in symptom severity. Thus, the symptom severity of ASD participants was assessed and its relationships with TBW size and SIFI susceptibility were examined.

## Methods

### Participants

The present study included 42 participants (21 with ASD and 21 TD individuals). Participants with ASD had been referred to Kyoto University by affiliated hospitals and public consultation offices for consultation or cognitive assessments, or by public organizations for employment evaluations. Because the TBW size of nine subjects (six individuals with ASD and three TD individuals) could not be calculated appropriately (see the “TOJ task” section), the data from 15 individuals with ASD (mean age ± SD: 28.13 ± 7.16 years; six females and nine males) and 18 TD individuals (29.00 ± 10.39 years; seven females and 11 males) were included in the main analysis. The demographic characteristics of participants are presented in Table 1. The ASD participants were diagnosed with Asperger’s disorder (*n* = 9) or pervasive developmental disorder, not otherwise specified (*n* = 6), by psychiatrists with expertise in developmental disorders according to Diagnostic and Statistical Manual for Mental Disorders-fourth edition-text revision (DSM-IV-TR) criteria (APA 2000). The diagnosis was based on interviews with participants and information from parents, professionals who had helped them and, when available, clinical records from childhood. Psychiatrists also assessed the symptom severity of the ASD participants using the Childhood Autism Rating Scale (CARS; Schopler et al. 1986) and the Childhood Autism Rating Scale, second edition, high-functioning version (CARS2-HF; Schopler et al. 2010). The CARS and CARS2-HF consist of 15 items addressing autism-related behaviors. The score of each item ranges from 1.0 to 4.0, and total scores range from 15.0 to 60.0. Higher scores indicate more severe symptoms. Although the average total score on the CARS (24.80 ± 3.36) was lower than the cut-off (27.0) for a diagnosis of autistic disorder (see Mesibov et al. 1989), the average total score on the CARS2-HF (30.03 ± 4.12) was higher than the cut-off for ASD (28.0; Schopler et al. 2010), indicating that the symptoms of the ASD participants were severe enough to warrant a diagnosis of ASD. We used CARS2-HF scores as a measure of symptom severity because the scores differed substantially among individuals. Exclusion criteria included history of a current psychotic disorder, substance or alcohol abuse, traumatic head injury, genetic disorder associated with autism, intellectual disability, or any other medical condition significantly affecting brain function (e.g., epilepsy).

**Table 1:** Demographic characteristics of ASD and TD groups

	ASD (*n* = 15) Mean (SD)	TD (*n* = 18) Mean (SD)	ASD versus TD		
			Statistics	*p*-value	Effect sizes
Age (years)	28.13 (7.16)	29.00 (10.39)	*t*(30.051) = − 0.283	0.779	*d* = 0.10
Sex (% male)	60.0%	61.1%	Fisher's exact test	1.000	
Full-scale IQ	112.33 (9.53)	113.89 (13.38)	*t*(31) = − 0.377	0.709	*d* = 0.13
Verbal IQ	117.67 (13.74)	112.94 (15.02)	*t*(31) = 0.934	0.357	*d* = 0.33
Performance IQ	102.67 (10.77)	112.06 (12.25)	*t*(31) = − 2.314	0.027	*d* = 0.81
CARS	24.80 (3.36)				
CARS2-HF	30.03 (4.12)				
AQ	32.07 (5.40)	16.11 (9.22)	*t*(28.104) = 6.177	< 0 .001	*d* = 2.11

*ASD* autism spectrum disorder, *TD* typical development, *IQ* intelligence quotient, *CARS* Childhood Autism Rating Scale, *CARS2-HF* Childhood Autism Rating Scale second edition, high functioning version, *AQ* Autism-Spectrum Quotient

The 21 TD participants were matched with 21 ASD participants in terms of age, sex, and full-scale IQ as assessed by the Japanese version of the Wechsler Adult Intelligence Scale, third edition (WAIS-III: Fujita et al. 2006; Wechsler 1997). Even when the nine subjects whose TBWs were difficult to calculate were excluded from the statistical analyses based on TOJ performance, the ASD (*n* = 15) and TD groups (*n* = 18) did not differ in terms of age (*t* [30.051] = − 0.283, *p* = 0.779), sex (*p* = 1.000), or full-scale IQ (ASD: 112.33 ± 9.53; TD: 113.89 ± 13.38; *t*[31] = − 0.377, *p* = 0.709). Although there were no significant differences between the ASD and TD groups in verbal IQ (ASD: 117.67 ± 13.74; TD: 112.94 ± 15.02; *t*[31] = 0.934, *p* = 0.357), the performance IQ (PIQ) of the TD group was significantly higher than that of the ASD group (ASD: 102.67 ± 10.77; TD: 112.06 ± 12.25; *t*[31] =  − 2.314, *p* = 0.027). All participants completed the Japanese version of the Autism Spectrum Quotient scale (AQ: Baron-Cohen et al. 2001; Wakabayashi et al. 2004), which consists of 50 self-rated items evaluating five domains (social skills, communication, imagination, attention to detail, and attention-switching). As expected, the average total score of the ASD group (32.07 ± 5.40) was significantly higher than that of the TD group (16.11 ± 9.22; *t*[28.104] = 6.177, *p* < 0.001). The average total score of the TD group was somewhat lower than that in a large study conducted in Japan (Wakabayashi et al. 2004; mean ± SD: 20.7 ± 6.4).

All experimental procedures were approved by the Ethics Committee of the Graduate School and Faculty of Medicine at Kyoto University and were performed in accordance with the ethical standards prescribed by the 1964 Declaration of Helsinki and its later amendments. All participants provided written informed consent for their participation in the study and received a gift card equivalent to 1000 yen per hour.

### Apparatus

Presentation 18.3 (NeuroBehavioral Systems, Albany, CA) implemented on a Windows computer (Optiplex 9020; Dell, Round Rock, TX) was used to control stimulus presentation and data acquisition. All visual stimuli were presented at eye level on a 23.5-inch monitor (FG2421; Eizo, Ishikawa, Japan) with a screen resolution of 1920 × 1080 pixels and a refresh rate of 120 Hz. All auditory stimuli were produced by headphones (MDR-1A; Sony, Tokyo, Japan). We confirmed the onset and duration of stimuli presentations using an USB oscilloscope (DrDAQ; Pico Technology, St. Neots, UK). All participants used a headrest to fix the distance between the monitor and their face at approximately 52 cm and responses were recorded via a keyboard.

### Procedure

The following experiments were conducted in a room without unnecessary noise and light. The order of the TOJ and SIFI tasks was counterbalanced.

### TOJ Task

**Fig. 1:**
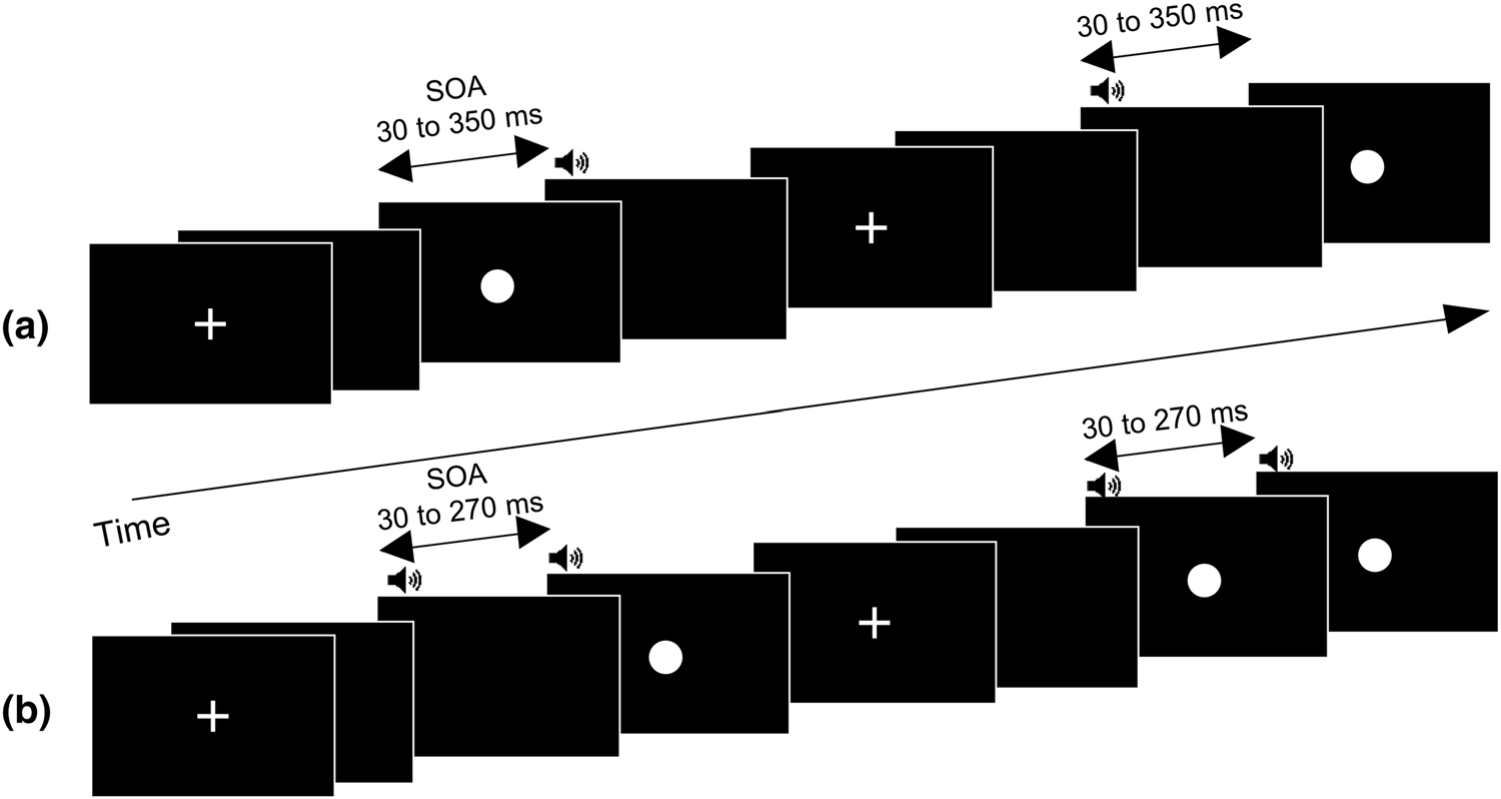
**a** Sequence of the temporal order judgment task. After presentation of a fixation cross, a white disk appeared against a black background; this was followed by a beep. In half the trials, the beep was followed by a flash after a variable delay (30–350 ms). In the other half of the trials, a flash was followed by a beep after a variable delay (30–350 ms). **b** Sequence of the sound-induced flash illusion task. This task included three different types of trial: unisensory, audio–visual-congruent, and illusory. In the illusory trials, after the presentation of a fixation cross, a single flash was presented with two beeps. One of the two beeps was always synchronized with the presentation of the flash, and the other beep either preceded or followed the bimodal stimulus pair (50% of the time for each)

We used the same experimental paradigm for the TOJ task used in a previous study (Kawakami et al. 2018; Fig. 1a). For each trial, a white fixation cross appeared on a black background at the center of the screen for 500–1500 ms; this was followed by an auditory stimulus and a visual stimulus. Participants were required to fixate on the cross and to judge whether a pure tone (3000 Hz, 80 dB, and 10 ms duration) appeared “early” or “late” compared to a white disk (diameter subtending a visual angle of 1.5° and 10 ms duration) by pressing one of two assigned keys (down arrow for sound early, right arrow for sound late) on a keyboard. No time limits were stipulated, and no feedback was given regarding the accuracy of the judgments in each trial. The SOAs between the visual and auditory stimuli were randomly varied (± 30, ± 70, ± 110, ± 150, ± 190, ± 230, ± 270, ± 310, and ± 350 ms; a negative value means the auditory stimulus was presented first). The task included 24 trials under each SOA condition, yielding a total of 432 trials. Participants were allowed to rest for a few minutes every 144 trials. Participants completed a training session with eight trials, and we confirmed that all participants understood the instructions.

We calculated the size of the TBW in which participants simultaneously perceived audio and visual information. Following previous studies (de Boer Schellekens et al. 2013; Kawakami et al. 2018), we performed a curve estimation for the percentage of flash-early responses under each SOA condition using a linear function as the principle function (mean *R*^*2*^ ± SD: 0.80 ± 0.20). We determined the TBW in which each participant could not accurately judge the order of the beep and flash by substituting 25% and 75% of the linear function for each individual; data from six subjects with ASD and three TD subjects were excluded from subsequent analyses because of their relatively low *R*^*2*^ values for the estimated curve (≤ 0.8 mean *R*^*2*^ ± SD = 0.54 ± 0.31). The calculated TBWs for the nine excluded subjects ranged from negative to positive values of the longest SOA conditions (mean TBW ± SD = 5650.9 ± 16746.5), which suggests that most of these subjects could not judge the order of the flash and beep even in the longest SOA trials. The *R*^*2*^ values for the remaining 15 subjects with ASD (mean *R*^*2*^ ± SD: 0.88 ± 0.03) and the 18 TD subjects (mean *R*^*2*^ ± SD: 0.87 ± 0.04) were sufficiently high. The *R*^*2*^ values of the ASD and TD groups did not differ (*t*[31] = 0.930, *p* = 0.360).

### SIFI Task

The present study used the same experimental paradigm for the SIFI task used in a previous study (Kawakami et al. 2018; Fig. 1b), which utilized a modified version of the one used by Setti et al. (2014). This task involves three types of trial: illusory, audio–visual-congruent, and unisensory. In the illusory trials, a flash appeared with two beeps; one beep was always presented at the same time as the flash, whereas the other beep either preceded or followed the bimodal stimulus pair (50% of the time for each type). The SOA between the beep and the bimodal stimulus pair was ± 30, 70, 110, 150, 190, 230, or 270 ms. The audio–visual-congruent trials involved two successive bimodal stimulus pairs with the same SOAs as those in the illusory trials. The uni-sensory trials included only one flash, only one beep, only two flashes, or only two beeps. A fixed SOA (70 ms) was used for the two-flashes-only and two-beeps-only trials. The flash and the beep were the same as those used in the TOJ task.

Participants were instructed to fixate on a central cross presented for 500–1500 ms and to respond to the number of perceived flashes by pressing one of three keys on a keyboard (a button labeled “1” for one flash, a button labeled “2” for two flashes, or a button labeled “0” for no flash). We treated the percentage of “two-flashes” responses in the illusory trials as reflective of the ability to engage in bimodal sensory integration. Trials with a response prior to the presentation of all stimuli were excluded from the analysis. With respect to each SOA condition, the task contained 24 trials under the unisensory and audio–visual-congruent condition and 12 trials under the illusory condition. In total, 432 trials were presented in a random order, and all participants took a short break after each set of 144 trials. To confirm that all participants understood the instructions, they completed a training session with seven trials before the testing session.

### Statistical Analysis

SPSS 25 (IBM Corp., Armonk, NY) was used for all statistical analyses. First, we examined group differences in TBW size and SIFI susceptibility. For the TOJ task, group differences in the proportion of trials in which participants reported seeing a flash first were examined using a repeated-measures analysis of variance (ANOVA), treating SOA as a within-subject factor and group as a between-subjects factor. When an interaction between group and SOA was significant, we performed a follow-up analysis to investigate the simple main effect of group under each SOA condition. When the sphericity assumption was violated, the degrees of freedom were corrected using the Greenhouse–Geisser correction. We used *t* tests to identify between-group differences in TBW size, based on an estimated function. The same procedure involving a repeated-measures ANOVA was followed for the SIFI task, and follow-up analyses were performed on the accuracy rate in the congruent trials and the proportion of two-flashes responses in the illusory trials. We also performed two-sample *t* tests on the accuracy rate in the unisensory trials to determine if the stimuli were realistically perceived. Although the PIQ scores differed significantly between groups, preliminary analyses on the TOJ and the SIFI tasks did not identify a significant main effect of this variable (*F*[1, 30] ≤ 1.177, *p* ≥ 0.287). Thus, we did not report the results of analyses using the PIQ as a covariate.

Next, relationships between temporal processing, audio–visual integration, and the symptoms of ASD were examined. In the ASD group, Pearson’s correlations between TBW size, SIFI susceptibility (i.e., the percentage of the responses for “two flashes” in the illusory trials across SOA conditions), and total score on the CARS2-HF were calculated. Then, their partial correlations were calculated, with sex, age, and PIQ included as covariates. In the TD group, the same analyses were applied to the correlations between TBW size, SIFI susceptibility, and total AQ score. Correlation analyses were also conducted across diagnostic groups. The alpha level was set at 0.05 for all statistical analyses, except the follow-up tests of repeated-measures ANOVAs with Bonferroni correction for multiple comparisons. Depending on the small sample sizes for examining correlations between different measures, a Bayes factor (BF_01_) was reported to allow more accurate interpretation of whether the evidence was for or against the null hypothesis. The BF_01_ is a ratio of the marginal likelihood of two competing hypotheses (null hypothesis to alternative hypothesis). According to the criterion of Jarosz and Wiley (2014), it was determined whether the evidence supported the alternative hypothesis (BF_01_ < 1) rather than the null hypothesis. Spearman’s rank-ordered correlations were also provided for each correlation analysis.

## Results

### Group Differences in TOJ Task

**Fig. 2:**
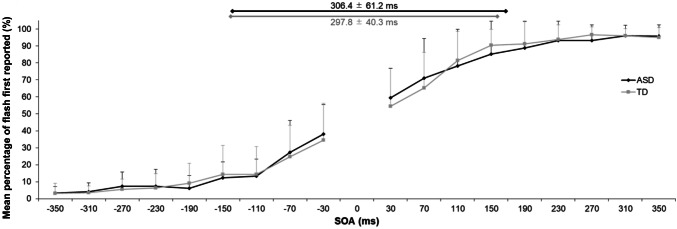
Mean percentage of the “flash-first” response under each stimulus onset asynchrony (SOA) condition in the temporal order judgement task. Error bars represent the standard deviation of the mean. The lines on the upper side of the graph represent the means of TBW size for each group. *ASD* autism spectrum disorder; *TD* typical development

Figure 2 presents the results of the TOJ task and shows the proportion of trials in which participants reported that the flash was first under each SOA condition. A repeated-measures ANOVA with SOA and group did not find a significant main effect of group (*F*[1, 30] < 0.001, *p* = 0.997, *η*_*p*_^*2*^ < 0.000) or an interaction between group and SOA (*F*[3.654, 113.270] = 0.501, *p* = 0.718, *η*_*p*_^*2*^ = 0.016). There was a significant main effect of SOA (*F*[3.654, 113.270] = 345.157, *p* < 0.001, *η*_*p*_^*2*^ = 0.918), indicating that the percentage of flash-early responses gradually became more positive as a function of increases in the SOA in both groups. There was no significant difference in TBW size between the ASD (mean ± SD: 306.4 ± 61.2 ms) and TD (mean ± SD: 297.8 ± 40.3 ms; *t*[31] = 0.485, *p* = 0.631, *d* = 0.17) groups.

### Group Differences in SIFI Task

There were no significant group differences in the accuracy rates (mean ± SD%) in the one-flash-only (ASD: 93.9 ± 11.2%; TD: 96.3 ± 3.8%; *t*[16.6] = − 0.794, *p* = 0.438, *d* = 0.30), two-flash (ASD: 85.6 ± 21.1%; TD: 89.4 ± 12.7%; *t*[31] = − 0.639, *p* = 0.528, *d* = 0.22), and one-beep-only trials (ASD: 99.7 ± 1.1%; TD: 99.1 ± 3.1%; *t*[31] = 0.782, *p* = 0.440, *d* = 0.25). The performances under all unisensory conditions were sufficiently high to suggest that participants with and without ASD almost always perceived the stimuli accurately.

**Fig. 3:**
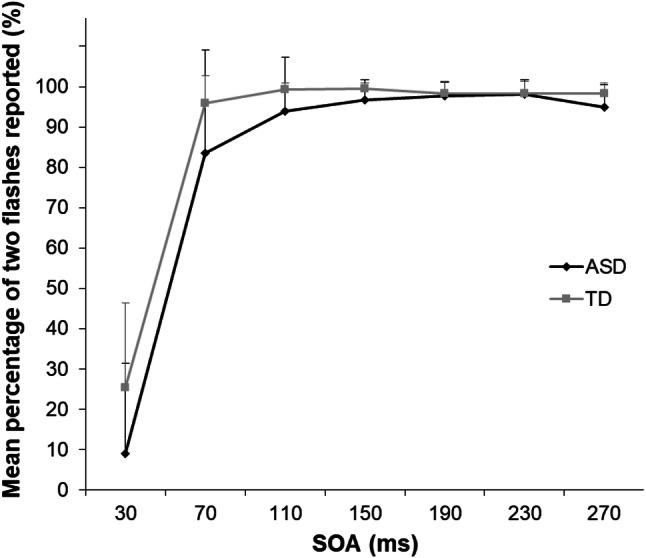
Mean percentage of the “two-flashes” response under each stimulus onset asynchrony (SOA) condition in the audio–visual-congruent trials of the sound-induced flash illusion task. Error bars represent the standard deviation of the mean. *ASD* autism spectrum disorder; *TD* typical development

Figure 3 shows the results for the audio–visual-congruent trials of the SIFI task. A repeated-measures ANOVA with SOA as a within-subject factor and group as a between-subjects factor revealed significant main effects of SOA (*F*[2.093, 64.873] = 246.830, *p* < 0.001, *η*_*p*_^*2*^ = 0.888) and group (*F*[1, 30] = 7.600, *p* = 0.010, *η*_*p*_^*2*^ = 0.197), which indicates that the TD group reported seeing two flashes more frequently than the ASD group when two flash-and-beep sets were presented. The interaction between group and SOA was not significant (*F* [2.093, 64.873] = 2.582, *p* = 0.081, *η*_*p*_^*2*^ = 0.077).

**Fig. 4:**
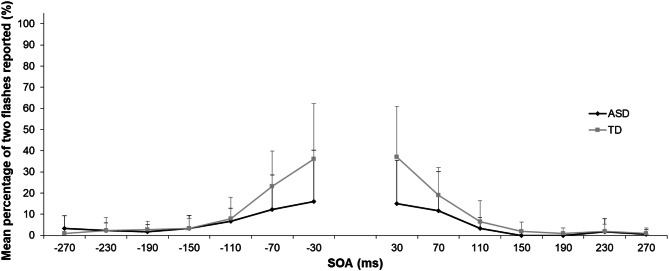
Mean percentage for the “two-flashes” response under each stimulus onset asynchrony (SOA) condition in the illusory trials of the sound-induced flash illusion task. Error bars represent the standard deviation of the mean. *ASD* autism spectrum disorder; *TD* typical development

The proportions of illusory trials in which participants reported two flashes are shown in Fig. 4. A repeated-measures ANOVA with SOA as a within-subject factor and group as a between-subjects factor found a significant main effect of group (*F*[1, 31] = 6.106, *p* = 0.019, *η*_*p*_^*2*^ = 0.165) and SOA (*F*[2.351, 72.885] = 22.719, *p* < 0.001, *η*_*p*_^*2*^ = 0.423) and an interaction between group and SOA (*F*[2.351, 72.885] = 3.827, *p* = 0.021, *η*_*p*_^*2*^ = 0.110). Follow-up analysis of the interaction did not reveal any significant group differences across SOA conditions after Bonferroni correction for multiple comparisons (α = 0.0036), although the ASD group was less likely than the TD group to perceive the SIFI under the − 30 (*F*[1, 31] = 4.823, *p* = 0.036) and 30-ms (*F*[1, 31] = 7.396, *p* = 0.011) SOA conditions.

### Correlations Between TBW Size and SIFI Susceptibility

The results of the correlation analyses between TBW size and SIFI susceptibility are presented in Table 2. There were no significant correlations in the TD group and the trends did not change even when the effects of sex, age, and PIQ were controlled for (all *p* > 0.553). The results of the Bayesian Pearson correlation analysis (hypothesis H_0_: TBW size is not correlated with SIFI susceptibility; H_1_: alternative hypothesis) showed a BF_01_ of 5.355 in the TD group, which provides moderate support for the hypothesis that TBW size was not correlated with SIFI susceptibility in the present study (cf. Jarosz and Wiley 2014).

**Table 2:** Correlations between TBW size and SIFI susceptibility

	ASD (*n* = 15)	TD (*n* = 18)	All participants (*n* = 33)
Pearson			
* r*	0.663 (0.007)	0.074 (0.771)	0.308 (0.081)
Partial *r*	0.542 (0.069)	0.156 (0.578)	0.343 (0.063)
Bayes factor			
BF_01_	0.140	5.355	1.630
Spearman			
* r*	0.456 (0.088)	0.134 (0.597)	0.284 (0.109)
Partial *r*	0.493 (0.103)	0.167 (0.553)	0.320 (0.084)

*TBW* temporal binding window, *SIFI* sound-induced flash illusion, *ASD* autism spectrum disorder, *TD* typical development, *Pearson* Pearson’s correlation, *Spearman* Spearman's rank-ordered correlation, control variables for the partial *r*: sex, age, and performance IQ, *p* values in parentheses, *BF*_*01*_ a ratio of the marginal likelihood of a null hypothesis to an alternative hypothesis

**Fig. 5:**
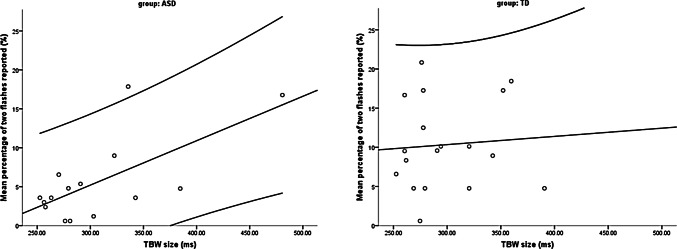
Scatterplots of the correlations between TBW size and SIFI susceptibility with regression lines and 95% confidence intervals for each group. *ASD* Autism spectrum disorder; *TD* typical development

In the ASD group, the Pearson’s correlation between TBW narrowness and low SIFI susceptibility was significant (*r* = 0.663, *p* = 0.007), which suggests that individuals with ASD, who have a narrow TBW, were less likely to perceive the SIFI (Fig. 5). When the effects of sex, age, and PIQ were controlled for, the trend for a correlation between TBW size and SIFI susceptibility did not markedly change (*r* = 0.542, *p* = 0.069). The results of a Bayesian Pearson correlation analysis (H_0_: TBW size is not correlated with SIFI susceptibility; H_1_: alternative hypothesis) showed a BF_01_ of 0.140 in the ASD group, which suggests that hypothesis H_1_ was 7.143 times more likely than H_0_. This finding provides moderate evidence for the hypothesis that TBW size was correlated with SIFI susceptibility in the present study (cf. Jarosz and Wiley 2014). Analysis of the relationship across groups (*r* = 0.308, *p* = 0.081) yielded a BF_01_ of 1.630, which provides anecdotal evidence for the hypothesis that TBW size was not correlated with SIFI susceptibility in the present study.

### Associations of Temporal Processing and Audio–Visual Integration with Symptom Severity in the ASD Group and with Autistic Traits in the TD Group

The results of the correlation analyses for symptom severity in the ASD group and autistic traits in the TD group are shown in Tables 3 and 4, respectively. In the ASD group, the correlation between symptom severity as measured by the CARS2-HF and SIFI susceptibility was not significant (*r* = 0.438, *p* = 0.103). When sex, age, and PIQ were entered as covariates, the result still did not reach significance (*r* = 0.558, *p* = 0.059). In the TD group, there were no significant correlations between AQ scores and performance on the TOJ and SIFI tasks (all *p* > 0.175), irrespective of whether the effects of sex, age, and PIQ were controlled for. Analyses across the diagnostic groups did not reveal any significant relationships between AQ score and performance on the TOJ and SIFI tasks (all *p* > 0.190). The results of the Bayesian Pearson correlation analysis (H_0_: symptom severity or autistic traits is/are not correlated with TBW size or SIFI susceptibility; H_1_: alternative hypothesis) support the null hypothesis, rather than the alternative hypothesis (all BF_01_ > 1).

**Table 3:** Correlations between TBW size and AQ scores or CARS2-HF scores

	CARS2-HF (ASD, *n* = 15)	AQ (TD, *n* = 18)	AQ (All, *n* = 33)
Pearson			
*r*	0.241 (0.388)	0.015 (0.952)	0.149 (0.408)
Partial *r*	0.393 (0.207)	0.107 (0.703)	0.056 (0.770)
Bayes factor			
BF_01_	3.539	5.576	5.272
Spearman			
*r*	0.079 (0.780)	0.177 (0.483)	0.110 (0.541)
Partial *r*	0.084 (0.796)	0.278 (0.192)	0.017 (0.930)

*TOJ* temporal order judgment, *AQ* autism-spectrum quotient, *CARS2-HF* Childhood Autism Rating Scale second edition, high-functioning version, *TBW* temporal binding window, *TD* typical development, *ASD* autism spectrum disorder, *Pearson* Pearson’s correlation, *Spearman* Spearman's rank-ordered correlation; control variables for the partial *r*: sex, age, and performance IQ, *p* values in parentheses, *BF*_*01*_ a ratio of the marginal likelihood of a null hypothesis to an alternative hypothesis

**Table 4:** Correlations between SIFI susceptibility and AQ scores or CARS2-HF scores

	CARS2-HF (ASD, *n* = 15)	AQ (TD, *n* = 18)	AQ (All, *n* = 33)
Pearson			
*r*	0.438 (0.103)	0.085 (0.737)	− .184 (0.305)
Partial *r*	0.558 (0.059)	0.076 (0.787)	− .246 (0.190)
Bayes factor			
BF_01_	1.358	5.280	4.395
Spearman			
*r*	0.370 (0.175)	0.355 (0.175)	− .164 (0.361)
Partial *r*	0.364 (0.245)	0.357 (0.192)	− .185 (0.327)

*SIFI* sound-induced flash illusion, *AQ* autism-spectrum quotient, *CARS2-HF* Childhood Autism Rating Scale second edition, high-functioning version, *TBW* temporal binding window, *TD* typical development, *ASD* autism spectrum disorder, *Pearson* Pearson’s correlation, *Spearman* Spearman's rank-ordered correlation, control variables for the partial *r*: sex, age, and performance IQ, *p* values in parenthesis, *BF*_*01*_ a ratio of the marginal likelihood of a null hypothesis to an alternative hypothesis

## Discussion

The present study used the SIFI and TOJ tasks to investigate whether multisensory integration and the temporal processing of audio–visual stimuli were atypical in high-functioning adults with ASD. Furthermore, based on a previous study in a general population (Kawakami et al. 2018), we tested the hypothesis that a narrower TBW was associated with lower SIFI susceptibility in a clinical population and that both features were prominent in individuals with more severe ASD symptoms.

The results showed that SIFI susceptibility was lower in the ASD group than the TD group. The results were not due to a failure to process the stimuli in each modality in the ASD group because both groups responded correctly to one flash-only trials and two flash-only trials. This finding is consistent with Stevenson et al. (2014), who found that children with ASD have lower SIFI susceptibility than TD children. Kawakami et al. (2018) also demonstrated that TD adults with high levels of autistic traits are less likely to perceive the SIFI under the same experimental paradigm used in the present study. These findings indicate that the visual perception of individuals with ASD or higher levels of autistic traits were less affected by auditory information, suggesting a difficulty in integrating audio–visual information. Consistent with this, a recent meta-analysis reported that people with ASD were less likely to experience the McGurk effect, an index of multisensory integration, compared with TD individuals (Zhang et al. 2019). Several studies have demonstrated that adults with ASD garnered less additional benefit from audio–visual information when distinguishing or identifying emotions (Charbonneau et al. 2013; Xavier et al. 2015). Similar to a previous study (Stevenson et al. 2014), the present study, which used a SIFI task consisting of flashes and beeps, extended the effects of the atypical multisensory integration associated with ASD into non-social domains.

However, there have been several inconsistent reports on SIFI susceptibility. Stevenson et al. (2018b) and van der Smagt et al. (2007) reported no significant differences in SIFI susceptibility between people with and without ASD. Foss-Feig et al. (2010) found higher SIFI susceptibility in children with ASD than in TD children. These inconsistent results may be explained by differences in the experimental paradigms used. First, the proportion of sets of flash-and-beep stimuli with a unisensory stimulus may have affected SIFI perception. The percentage of flash-and-beep sets in a study showing lower SIFI susceptibility in the ASD group (Stevenson et al. 2014) was lower than those in other studies reporting higher or equal SIFI susceptibility in this group (Foss-Feig et al. 2010; Stevenson et al. 2018b; van der Smagt et al. 2007). The percentage of flash-and-beep stimuli in the present study was closer to the former study. This suggest that, in cases of a low frequency of a flash accompanied by a beep, individuals with ASD are less likely to perceive an illusory flash. According to the view that individuals with ASD give much weight to subtle prediction errors to update a model of the environment (Van de Cruys et al. 2014), they may be easily influenced by the low proportion of flash-and-beep stimuli sets and may not have a stable model of a beep accompanied by a flash. Second, the present study did not include a condition with two flashes and one beep. Bao et al. (2017) employed a SIFI task that included fusion illusion (i.e., two flashes with one beep perceived as one flash) and reported that individuals with ASD experience the fusion illusion more frequently than TD participants, whereas there was no significant group difference for fission illusion (i.e., one flash with two beeps perceived as two flashes). Other studies that reported non-significant differences in fission illusion have included a condition with two flashes and one beep (Foss-Feig et al. 2010; Stevenson et al. 2018b; van der Smagt et al. 2007) whereas studies showing lower fission illusion susceptibility in the ASD group did not include a fusion illusion condition (Stevenson et al. 2014; Kawakami et al. 2018). A fusion illusion condition inducing perception of a flash accompanied by a beep might make the perception of an illusory flash under the fission illusion more likely in individuals with ASD. The conflicting findings might reflect the differential effects of contextual information (i.e., the frequency of a flash accompanied by a beep) on multisensory integration between people with and without ASD; these differences would depend on the degree of sensitivity to deviations from predictions (cf. Van de Cruys et al. 2014).

A group difference was also found in congruent trials. When two flash-and-beep sets were presented, the TD group reported seeing two flashes more frequently than did the ASD group. Consistent with the low SIFI susceptibility, suggestive of multisensory integration difficulties of individuals with ASD, this result implies that congruent auditory information provided a clue about the presence of two flashes to TD but not to ASD individuals. In contrast, it is possible that there was a response bias to the two-flash report that affected performance. In fact, there was a positive correlation between the percentage of two flashes reported in congruent trials and that reported in illusory trials (*r* = 0.627, *p* < 0.001, *n* = 33). This finding suggests that a bias for reporting two flashes in the TD group whenever a beep was paired with a flash superficially increase SIFI susceptibility. However, if this response bias occurred, the percentage of two-flash reports should be higher in the congruent condition than the illusion condition because two flashes were actually presented under the congruent condition. The results showed that the opposite pattern occurred in the shortest SOA condition. To evaluate the existence of the response bias itself, it would be better to consider performance in the one flash-only trials. In the present study, the two groups did not differ in their performances in the one flash-only trials. The task constructions in the present study also aimed to prevent this type of response bias. There were just two types of bi-modal conditions, the congruent condition (i.e., two flashes with two beeps) and the illusory condition (i.e., one flash with two beeps), and the number of trials for each condition was the same.

It is also possible that the auditory information distracted individuals with ASD from processing the visual information. This interpretation raises a question about whether the low frequency of two-flash reports from the ASD group under illusory trials was caused by difficulties with temporal discrimination of the flashes (a real and an illusory flash) when accompanied by auditory information rather than by difficulties in multisensory integration. However, under the 30-ms SOA condition, a higher percentage of two-flash reports were provided in illusory than congruent trials in both groups (see Figs. 3,4), although only one flash was presented under the illusory trials. The data suggest that reduced SIFI perception was induced by problems with multisensory integration rather than by issues with temporal discrimination arising from intolerance of other modal interruptions.

The present study also found that TBW size was positively associated with SIFI susceptibility in the ASD group, i.e., people with ASD, who have a narrow TBW, were less likely to perceive the SIFI. This result is consistent with those of previous studies in a general population (Stevenson et al. 2012; Kawakami et al. 2018). These findings suggest that, although the present study did not find a relationship between TBW size and SIFI susceptibility in the TD group, the relationship between TBW size and multisensory integration that has been reported in general populations (Stevenson et al. 2012, 2018b; Kawakami et al. 2018) also holds true in adults with ASD. Previous studies have proposed that the TBW determines which information should be integrated because it is more likely that information received close in time would originate from the same source (Kawakami et al. 2018; Stevenson et al. 2012; Wallace and Stevenson 2014). A too-narrow TBW renders it less likely that multisensory information would be perceived as being presented simultaneously and arising from the same event. Although high temporal resolution is typically helpful under multisensory and unisensory processing, such as speech perception (e.g., Stevenson et al. 2018a) and musical expertise (e.g., Kühnis et al. 2013), this may prevent the integration of multisensory information that would ordinarily occur with a subtle temporal gap such as one flash with two beeps trials. The SIFI is considered to occur when an individual predicts that a beep will accompany a flash (Chan et al. 2016). People whose TBW is narrow may more easily notice a beep without a flash (i.e., single flash in an illusory trial) under the shorter SOA condition. Based on this prediction error, they may reject the prediction that a beep will accompany a flash, thereby exhibiting low SIFI susceptibility. Together with previous studies (Stevenson et al. 2012; Kawakami et al. 2018), the present study suggests that these underlying multisensory integration processes are shared by individuals with and without ASD.

Although de Boer-Schellekens et al. (2013) found differences between the performances of adolescents with and without ASD on the TOJ task, we found no significant differences between the ASD and TD groups in this regard. This discrepancy may relate to the developmental changes that occur during and after adolescence (cf. Casassus et al. 2019). Indeed, Stevenson et al. (2018a) found that the temporal acuity for audio–visual information improved until around middle age and then declined with age in older individuals. If the developmental change in the TBW follows a quadratic function, there should be a period in which there is no detectable group difference despite the existence of developmental delay in ASD. As the age range of our sample was wide (ASD: 28.13 ± 7.16 years; TD: 29.00 ± 10.39 years), it is possible that it included the peak of the narrowed TBW in both groups, leading to the ostensible equivalence in this variable.

The present study has some additional limitations. First, it included a small sample size, particularly for the ASD group, because some individuals with ASD had difficulty performing the TOJ task. Thus, the results should be interpreted with caution. Although we did not find a group difference in TBW, this may have been due to lack of statistical power. Indeed, post-hoc power analyses showed that the statistical powers were low for non-significant correlations (< 0.4) and a previous finding regarding significant relationships among autistic traits, TBW size, and SIFI susceptibility in the TD group (Kawakami et al. 2018) was not replicated in the present study. It is possible that a higher-powered study would have observed these effects. Studies using a less demanding task and a larger sample will be needed to investigate the TBW in individuals with ASD. In addition, due to the difficulty of the TOJ task, nine subjects (six subjects with ASD and three with TD) were excluded from the statistical analyses. Future studies should consider the alteration in the processing of sensory information across groups. Second, methodological limitations may have influenced the results. In the present study, auditory and visual stimuli were presented in different spatial positions, which enabled both groups to distinguish the order of the flash and beep (cf. Zampini et al. 2003). Thus, it is possible that group differences in TBW might have been obscured. Third, although the developmental pattern of the TBW was discussed based on a literature review, almost all the present participants were young adults. Direct comparisons of the TBW and multisensory integration in child, adolescent, and adult groups are needed to confirm the present speculations. Fourth, the present study did not investigate the effects of the TBW and the ability to engage in multisensory integration on social–cognitive functioning in ASD. Although the present study did not find any associations between symptom severity and the TBW or SIFI susceptibility, the CARS total score did not directly reflect a specific aspect of social cognition. A promising approach would involve investigation of the influence of these fundamental functions on performance of a social–cognitive task requiring temporal integration, such as the processing of dynamic facial expressions (e.g., Uono et al. 2014).

In summary, the present study showed that the ASD group was less likely than the TD group to perceive an illusory flash induced by multisensory integration during the SIFI task. The correlation and Bayesian analyses provided moderate evidence that the reduced SIFI susceptibility was associated with the narrow TBW in the ASD group, although the groups showed similar TBWs. These results suggest that the individuals with ASD exhibited atypical multisensory integration, and that individual differences in the efficacy of this process may be affected by the temporal processing of multisensory information.
